# An increase in the medial proximal tibial angle of 95° or greater after opening wedge high tibial osteotomy does not necessarily lead to excessive knee joint line obliquity of 5° or greater

**DOI:** 10.1051/sicotj/2026028

**Published:** 2026-05-19

**Authors:** Yuta Hizume, Ken Kumagai, Tomotaka Akamatsu, Shuntaro Nejima, Yusuke Inoue, Hyonmin Choe, Hiroyuki Ike, Naomi Kobayashi, Yutaka Inaba

**Affiliations:** 1 Department of Orthopaedic Surgery, Graduate School of Medicine, Yokohama City University 3-9 Fukuura, Kanazawa-ku Yokohama 236-0004 Japan; 2 Department of Orthopaedic Surgery, Yokohama City University Medical Center 4-57 Urafune-cho, Minami-ku Yokohama 232-0024 Japan

**Keywords:** Opening wedge high tibial osteotomy, Medial proximal tibial angle, Knee joint line obliquity, Ankle joint line obliquity, Radiographic assessment

## Abstract

*Introduction*: The relationship between medial proximal tibial angle (MPTA) overcorrection and knee joint line obliquity (KJLO) after opening wedge high tibial osteotomy (OWHTO) remains controversial. Though excessive valgus correction (MPTA ≥ 95°) has been associated with abnormal joint line inclination, the extent to which this affects postoperative alignment and whether overcorrection necessarily results in excessive KJLO (≥5°), which has been associated with increased shear stress on the articular cartilage, is unclear. *Methods*: A total of 300 knees undergoing isolated OWHTO were retrospectively reviewed. Postoperative KJLO and MPTA were measured using full-length standing radiographs. Knees were stratified into MPTA <95° and ≥95° groups. Correlations between postoperative MPTA and KJLO were analyzed, and in the overcorrected (MPTA ≥ 95°) group, logistic regression and receiver-operating characteristic (ROC) curve analyses were performed to identify preoperative predictors of postoperative KJLO ≥ 5°. *Results*: Postoperative MPTA showed a significant positive correlation with KJLO (*r* = 0.61, *p* < 0.001). However, 52.5% of knees with MPTA ≥ 95° maintained KJLO < 5°, indicating that overcorrection does not necessarily cause excessive joint line inclination. Multivariable analysis identified preoperative lateral distal femoral angle (LDFA), knee joint line convergence angle (KJLCA), and ankle joint line obliquity (AJLO) as independent predictors of postoperative KJLO ≥ 5° in knees with MPTA ≥ 95° (*p* < 0.01). ROC curve analyses showed that AJLO ≥ 5.6°, LDFA ≥ 88.9°, and KJLCA ≥ 4.5° were the cutoff values predicting KJLO ≥ 5°. *Conclusions*: Although postoperative MPTA correlates with KJLO, more than half of the overcorrected cases maintained acceptable joint line inclination. Preoperative AJLO, LDFA, and KJLCA are key parameters for predicting postoperative KJLO ≥ 5° in knees with MPTA ≥ 95°, highlighting the importance of comprehensive preoperative alignment assessment when planning OWHTO.

## Introduction

Opening wedge high tibial osteotomy (OWHTO) is a widely accepted surgical procedure for treating osteoarthritis (OA) of the knee and spontaneous osteonecrosis of the knee (SONK). By shifting the mechanical axis laterally, this procedure reduces the load on the medial compartment and alleviates symptoms, thereby preserving the native joint and delaying the need for arthroplasty [1–3].

Although OWHTO provides reliable clinical benefits, concerns have been raised regarding the excessive increase in the medial proximal tibial angle (MPTA) after surgery. Previous studies have suggested that overcorrection with an MPTA ≥95° may result in abnormal knee joint line obliquity (KJLO) and potentially compromise long-term outcomes [4–6]. A KJLO ≥5°, defined as excessive KJLO, is generally considered beyond the acceptable range, since it may increase in shear stress on the articular cartilage [7]. However, the evidence regarding its actual effects on clinical results has been inconsistent, and the long-term relationships between overcorrected MPTA and patient outcomes remain controversial [8].

Recent investigations have suggested that even with MPTA ≥95°, clinical outcomes are not necessarily compromised, possibly due to compensatory changes in the hip and ankle joints that mitigate the effect of the increased KJLO [9–11]. Although these compensatory mechanisms explain why overcorrection does not uniformly worsen results, the relationship between the magnitude of the MPTA increase and the degree of KJLO change has not been fully elucidated. The predictability of cases in which KJLO remains within an acceptable range despite a marked increase in MPTA has not been clarified.

The aim of this study was to investigate the relationship between postoperative changes in MPTA and KJLO and to explore which proportion of cases with an overcorrected MPTA (≥95°) still present with a KJLO within the acceptable range of <5°. Furthermore, this study sought to identify preoperative radiographic factors associated with excessive postoperative KJLO (≥5°) in the overcorrected cases with MPTA ≥95°. We hypothesized that postoperative MPTA ≥95° does not necessarily result in excessive KJLO (≥5°), and that specific preoperative alignment parameters would independently predict the development of excessive KJLO.

## Materials and methods

### Patients

A total of 318 knees from 290 patients with knee OA or SONK who underwent isolated OWHTO using a TomoFix plate (DePuy Synthes, Zuchwil, Switzerland) from 2014 to 2023 were investigated retrospectively. The inclusion criteria were painful OA or SONK localized in the medial compartment of the knee and follow-up for more than 1 year, without any limitations based on age. The exclusion criteria encompassed individuals with significant varus deformity characterized by mechanical varus alignment exceeding 10°, mechanical valgus alignment, flexion contracture >15°, or those with a history of inflammatory arthritis, joint infection, or treatment with immunosuppressive therapy. Of the 318 knees reviewed in the study, 18 were excluded from the analysis: 16 were lost to follow-up, and two had correction loss due to lateral hinge fracture. A total of 300 knees from 276 patients were available for the analysis. The protocol and publication of the study were approved by our institutional review board. All participants in the study provided their written informed consent.

### Surgical procedure and postoperative management

OWHTO was performed via an anteromedial incision under fluoroscopic control [3]. The mechanical axis was adjusted to pass through a point located at 65% of the tibial Plateau width from the medial edge. The osteotomy was initiated approximately 35 mm below the medial joint line, using a biplanar technique. The oblique cut was directed from the medial cortex toward the upper one-third of the proximal tibiofibular joint, preserving the tibial tuberosity within the distal segment. After creating the osteotomy gap, the opening was gradually expanded, and two wedge-shaped β-tricalcium phosphate blocks with 60% porosity (Osferion; Olympus Terumo Biomaterials, Tokyo, Japan) were inserted. Fixation was achieved using a TomoFix locking plate.

Postoperatively, the patients began a structured rehabilitation program. Isometric quadriceps exercises and gentle range-of-motion training began on the first postoperative day. Patients were instructed to remain non-weight-bearing for 1 week, followed by progressive partial loading. Full weight-bearing was permitted after 2 weeks. The rehabilitation program emphasized continued isometric strengthening of the quadriceps, gluteal, and hamstring muscles, along with active and passive knee mobilization, patellar gliding, and stretching of the hamstrings and gastrocnemius-soleus complex. Walking with aids was encouraged during the early phase, advancing to independent ambulation and stair-climbing practice as tolerated.

### Assessment of radiographic outcomes

**Figure 1:**
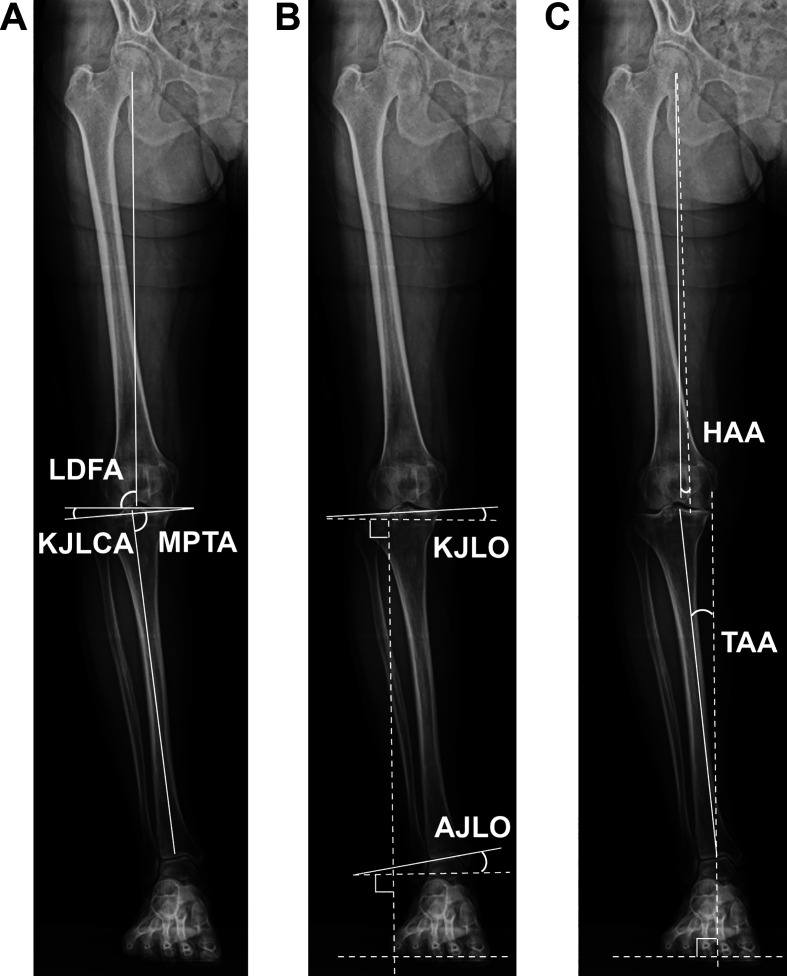
Radiographic measurements. (A) Lateral distal femoral angle (LDFA), medial proximal tibial angle (MPTA), and knee joint line convergency angle (KJLCA). (B) Knee joint line obliquity (KJLO) and ankle joint line obliquity (AJLO). (C) Hip abduction angle (HAA) and tibial axis angle (TAA).

Radiographic evaluation was conducted using standardized full-length, weight-bearing, anteroposterior radiographs of the lower extremity, obtained with patients standing upright and the patella facing forward to minimize rotational variability (Figure 1). The weight-bearing line ratio (WBLR) was calculated as the ratio of the distance from the medial edge of the proximal tibia to the passing point of the mechanical axis on the proximal tibia to the width of the proximal tibia. The lateral distal femoral angle (LDFA) was measured as the lateral angle formed between the femoral mechanical axis and the knee joint line of the femur. The MPTA was measured as the medial angle formed between the tibial mechanical axis and the knee joint line of the tibia. The knee joint line convergence angle (KJLCA) was measured as the angle formed between a line tangent to the distal femoral condyle and the proximal tibial Plateau. The KJLO was measured as the angle formed between the line parallel to the ground and the line tangential to the tibial plateau (positive value indicating lateral joint line inclination). The ankle joint line obliquity (AJLO) was measured as the angle formed between the line parallel to the ground and the line tangential to the talar dome (positive value indicating lateral joint line inclination). The hip abduction angle (HAA) was measured as the angle formed between the line perpendicular to the ground and the femoral mechanical axis. The tibial axis angle (TAA) was measured as the angle formed between the line perpendicular to the ground and the tibial mechanical axis [12]. Two experienced orthopedic surgeons (Y.H. and K.K.) independently conducted radiographic measurements on two separate occasions, more than 2 months apart.

### Statistical analysis

Statistical analysis was carried out using BellCurve for Excel version 4.09 (Social Survey Research Information Co., Ltd., Tokyo, Japan). Since histograms of the data showed that the variables had non-normal distributions, nonparametric statistical methods were used to analyze the data. The Mann–Whitney U test was used to test for significant differences between two groups. Pearson’s χ^2^ test was used to test for significant differences in the distributions of categorical variables. Multivariable logistic regression analysis was used to identify factors associated with postoperative KJLO ≥5°. Receiver-operating characteristic (ROC) curve analysis was used to determine the cutoff values of preoperative factors associated with postoperative KJLO ≥5°. An adjusted *p*-value <0.05 was considered significant. The intra- and inter-rater reliabilities of radiographic measurements were assessed by interclass correlation coefficients (ICCs).

## Results

### Patients’ characteristics

A total of 300 knees were analyzed, including 178 knees with a postoperative MPTA <95° and 122 knees with an MPTA ≥95°. No significant differences were observed between the two groups in age, sex, or body mass index (Table 1). However, the preoperatively planned correction angle was significantly greater in the MPTA ≥95° group than in the MPTA <95° group (12.5° ± 3.1° vs. 8.5° ± 2.9°, *p* < 0.001).

**Table 1:** Comparison of radiographic parameters between knees with MPTA <95° and ≥95° after opening wedge high tibial osteotomy.

Variable	Overall	MPTA < 95°	MPTA ≥ 95°	*p*-value
	(*n* = 300)	(*n* = 178)	(*n* = 122)	
Age, y	66.9 ± 8.6	66.3 ± 8.9	67.8 ± 8.2	0.210
Male/female, *n*	100/200	62/116	38/84	0.506
Body mass index, kg/m^2^	25.9 ± 4.0	25.8 ± 4.1	26.1 ± 3.9	0.328
Preoperatively planned correction angle, °	10.1 ± 3.6	8.5 ± 2.9	12.5 ± 3.1	**< 0.001**
Preoperative WBLR, %	18.0 ± 13.9	19.9 ± 12.5	15.1 ± 15.2	**0.002**
Preoperative LDFA, °	87.9 ± 2.3	87.3 ± 2.2	88.7 ± 2.2	**< 0.001**
Preoperative MPTA, °	84.3 ± 2.6	83.8 ± 2.4	85.0 ± 2.8	**< 0.001**
Preoperative KJLCA, °	3.6 ± 2.2	3.2 ± 2.0	4.1 ± 2.3	**< 0.001**
Preoperative KJLO, °	−0.1 ± 2.9	−1.0 ± 1.3	1.3 ± 2.9	**< 0.001**
Preoperative AJLO, °	7.0 ± 4.5	6.7 ± 4.1	7.5 ± 5.0	0.121
Preoperative HAA, °	1.5 ± 2.4	1.5 ± 2.4	1.6 ± 2.4	0.549
Preoperative TAA, °	5.6 ± 3.0	5.2 ± 2.6	6.2 ± 3.5	**0.009**
Postoperative WBLR, %	66.5 ± 12.4	62.9 ± 11.7	71.8 ± 11.6	**< 0.001**
Postoperative MPTA, °	94.4 ± 3.4	92.2 ± 2.1	97.6 ± 2.2	**< 0.001**
Postoperative KJLCA, °	2.4 ± 1.9	2.0 ± 1.8	2.9 ± 1.9	**< 0.001**
Postoperative KJLO, °	2.8 ± 3.1	1.5 ± 2.5	4.8 ± 2.7	**< 0.001**
Postoperative KJLO ≥ 5°, n (%)	77 (25.7)	19 (10.7)	58 (47.5)	**< 0.001**
Postoperative AJLO, °	−0.4 ± 4.5	0.0 ± 4.2	−0.1 ± 4.7	0.088
Postoperative HAA, °	−3.0 ± 2.3	−2.5 ± 2.3	−3.8 ± 2.2	**< 0.001**
Postoperative TAA, °	−1.6 ± 2.9	−0.8 ± 2.7	−2.8 ± 2.7	**< 0.001**

Mean ± standard deviation values are shown for continuous variables.

WBLR, weight-bearing line ratio; LDFA, lateral distal femoral angle; MPTA, medial proximal tibial angle; KJLCA, knee joint line convergence angle; KJLO, knee joint line obliquity; AJLO, ankle joint line obliquity; HAA, hip abduction angle; TAA, tibial axis angle.

### Radiographic findings

**Figure 2:**
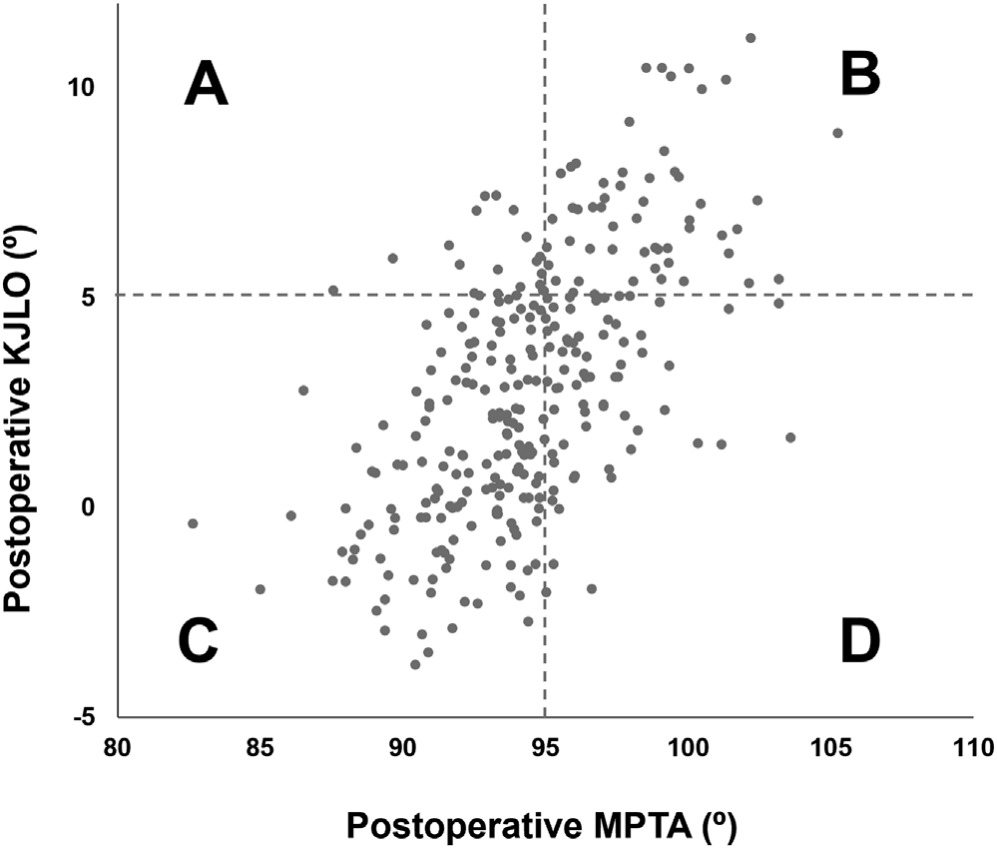
Distribution of postoperative medial proximal tibial angle (MPTA) and postoperative knee joint line obliquity (KJLO). The scatter plot shows a moderate correlation between postoperative MPTA and postoperative KJLO (*r* = 0.61, *p* < 0.001). A) KJLO ≥5°, MPTA <95°, *n* = 21. B) KJLO ≥5°, MPTA ≥95°, *n* = 58. C) KJLO <5°, MPTA <95°, *n* = 158. D) KJLO <5°, MPTA ≥95°, *n* = 64.

Preoperatively, the MPTA ≥95° group showed significantly smaller WBLR than the MPTA <95° group (15.1 ± 15.2% vs. 19.9 ± 12.5%, *p* < 0.01). The preoperative LDFA, MPTA, KJLCA, KJLO, and TAA were all significantly greater in the MPTA ≥95° group than in the MPTA <95° group (all *p* < 0.01). Postoperatively, the MPTA ≥95° group demonstrated significantly greater WBLR than the MPTA <95° group (71.8 ± 11.6% vs. 62.9 ± 11.7%, *p* < 0.001), indicating more valgus correction. Postoperative KJLO was also significantly higher in the MPTA ≥95° group than in the MPTA <95° group (4.8° ± 2.7° vs. 1.5° ± 2.5°, *p* < 0.001), and the proportion of knees with KJLO ≥5° was markedly greater in the MPTA ≥95° group than in the MPTA <95° group (47.5% vs. 10.7%, *p* < 0.001). Conversely, there were no significant intergroup differences in postoperative AJLO values (−0.1° ± 4.7° vs. 0.0° ± 4.2°, *p* = 0.088). A significant positive correlation was observed between postoperative MPTA and postoperative KJLO (*r* = 0.61, *p* < 0.001, Figure 2). However, even in knees with postoperative MPTA ≥95°, 52.5% (64 of 122 knees) exhibited KJLO <5°, indicating that an overcorrected tibial angle does not necessarily result in excessive knee joint line inclination. The ICCs for inter-rater and intra-rater reliabilities of radiographic measurements were 0.93 to 0.96 and 0.97 to 0.99, respectively, indicating good reliability.

### Comparison between KJLO <5° and ≥5° in patients with MPTA ≥95°

To further investigate the factors associated with excessive KJLO and an overcorrected MPTA, patients with MPTA ≥95° were subdivided into two groups according to postoperative KJLO <5° (*n* = 64) and ≥5° (*n* = 58) (Table 2). The knees with postoperative KJLO ≥5° had significantly smaller preoperative WBLR than the knees with KJLO <5° (11.1 ± 14.5% vs. 18.6 ± 15.0%, *p* < 0.001), suggesting more pronounced preoperative varus alignment. Preoperative TAA, LDFA, KJLCA, KJLO, and AJLO were all significantly greater in the knees with postoperative KJLO ≥5° than in the knees with postoperative KJLO <5° (all *p* < 0.01). Postoperatively, the knees with KJLO ≥5° showed higher MPTA, greater KJLCA, and smaller HAA than the knees with KJLO <5° (98.3° ± 2.2° vs. 97.1° ± 1.9°, *p* < 0.001; 3.6° ± 1.9° vs. 2.3° ± 1.8°, *p* < 0.001; −4.8° ± 1.9° vs. –2.8° ± 1.9°, *p* < 0.001, respectively). Notably, postoperative AJLO showed an opposite inclination between the knees with postoperative KJLO ≥5° and <5° (1.4° ± 3.9° vs. –3.1° ± 4.4°, *p* < 0.001).

**Table 2:** Comparison of radiographic parameters between KJLO ≥5° and <5° in knees with MPTA ≥95° after opening wedge high tibial osteotomy.

Variable	KJLO <5°	KJLO ≥5°	*p* value
	(*n* = 64)	(*n* = 58)	
Age, y	66.6 ± 7.9	69.2 ± 8.4	0.121
Male/female, n	20 / 44	18 / 40	0.979
Body mass index, kg/m^2^	26.3 ± 3.6	25.8 ± 4.3	0.292
Preoperatively planned correction angle, °	12.0 ± 3.2	13.1 ± 2.8	**0.022**
Preoperative WBLR, %	18.6 ± 15.0	11.1 ± 14.5	**< 0.001**
Preoperative LDFA, °	88.1 ± 2.3	89.4 ± 2.0	**< 0.001**
Preoperative MPTA, °	85.0 ± 3.0	85.1 ± 2.6	0.954
Preoperative KJLCA, °	3.5 ± 2.4	4.8 ± 1.9	**0.001**
Preoperative KJLO, °	0.2 ± 2.5	2.4 ± 2.8	**< 0.001**
Preoperative AJLO, °	5.5 ± 4.9	9.7 ± 4.0	**< 0.001**
Preoperative HAA, °	1.4 ± 2.4	1.8 ± 2.5	0.404
Preoperative TAA, °	5.2 ± 3.3	7.3 ± 3.4	**< 0.001**
Postoperative WBLR, %	73.5 ± 12.8	71.1 ± 11.3	0.177
Postoperative MPTA, °	97.1 ± 1.9	98.3 ± 2.2	**< 0.001**
Postoperative KJLCA, °	2.3 ± 1.8	3.6 ± 1.9	**< 0.001**
Postoperative KJLO, °	2.8 ± 1.7	7.1 ± 1.6	**< 0.001**
Postoperative AJLO, °	−3.1 ± 4.4	1.4 ± 3.9	**< 0.001**
Postoperative HAA, °	−2.8 ± 1.9	−4.8 ± 1.9	**< 0.001**
Postoperative TAA, °	−4.1 ± 2.3	−1.2 ± 2.3	**< 0.001**

Mean ± standard deviation values are shown for continuous variables.

WBLR, weight-bearing line ratio; LDFA, lateral distal femoral angle; MPTA, medial proximal tibial angle; KJLCA, knee joint line convergence angle; KJLO, knee joint line obliquity; AJLO, ankle joint line obliquity; HAA, hip abduction angle; TAA, tibial axis angle.

### Multivariable logistic regression analysis for preoperative factors associated with postoperative KJLO ≥5°

Multivariable logistic regression analysis identified preoperative LDFA (odds ratio (OR) = 1.76, 95% CI 1.21–2.56, *p* = 0.003), preoperative KJLCA (OR = 1.88, 95% CI 1.25–2.81, *p* = 0.002), and preoperative AJLO (OR = 1.21, 95% CI 1.06–1.39, *p* = 0.005) as independent predictors of postoperative KJLO ≥5° in knees with MPTA ≥95° after OWHTO (Table 3).

**Table 3:** Multivariable logistic regression of preoperative parameters associated with KJLO ≥5° in knees with MPTA ≥95° after opening wedge high tibial osteotomy.

Independent variable	*B* (95% CI)	Odds ratio (95% CI)	*p*-value
Age	0.027 (−0.037 – 0.091)	1.027 (0.963 – 1.096)	0.412
Sex	−0.866 (−1.992 – 0.261)	0.421 (0.136 – 1.298)	0.132
Body mass index	−0.136 (−0.273 – 0.002)	0.874 (0.762 – 1.001)	0.053
Preoperatively planned correction angle	0.137 (−0.109 – 0.384)	1.147 (0.896 – 1.468)	0.276
Preoperative WBLR	0.057 (−0.005 – 0.119)	1.059 (0.995 – 1.127)	0.074
Preoperative LDFA	0.565 (0.188 – 0.942)	1.759 (1.207 – 2.564)	**0.003**
Preoperative KJLCA	0.630 (0.226 – 1.034)	1.878 (1.254 – 2.812)	**0.002**
Preoperative KJLO	0.076 (−0.322 – 0.474)	1.079 (0.724 – 1.607)	0.708
Preoperative AJLO	0.194 (0.059 – 0.328)	1.213 (1.061 – 1.388)	**0.005**
Preoperative TAA	−0.014 (−0.330 – 0.303)	0.986 (0.719 – 1.354)	0.932

CI, confidence interval; WBLR, weight-bearing line ratio; LDFA, lateral distal femoral angle; KJLCA, knee joint line convergence angle; KJLO, knee joint line obliquity; AJLO, ankle joint line obliquity; TAA, tibial axis angle.

### Receiver-operating characteristic curve analyses using preoperative radiographic parameters for prediction of postoperative KJLO ≥5° in knees with MPTA ≥95°

**Figure 3:**
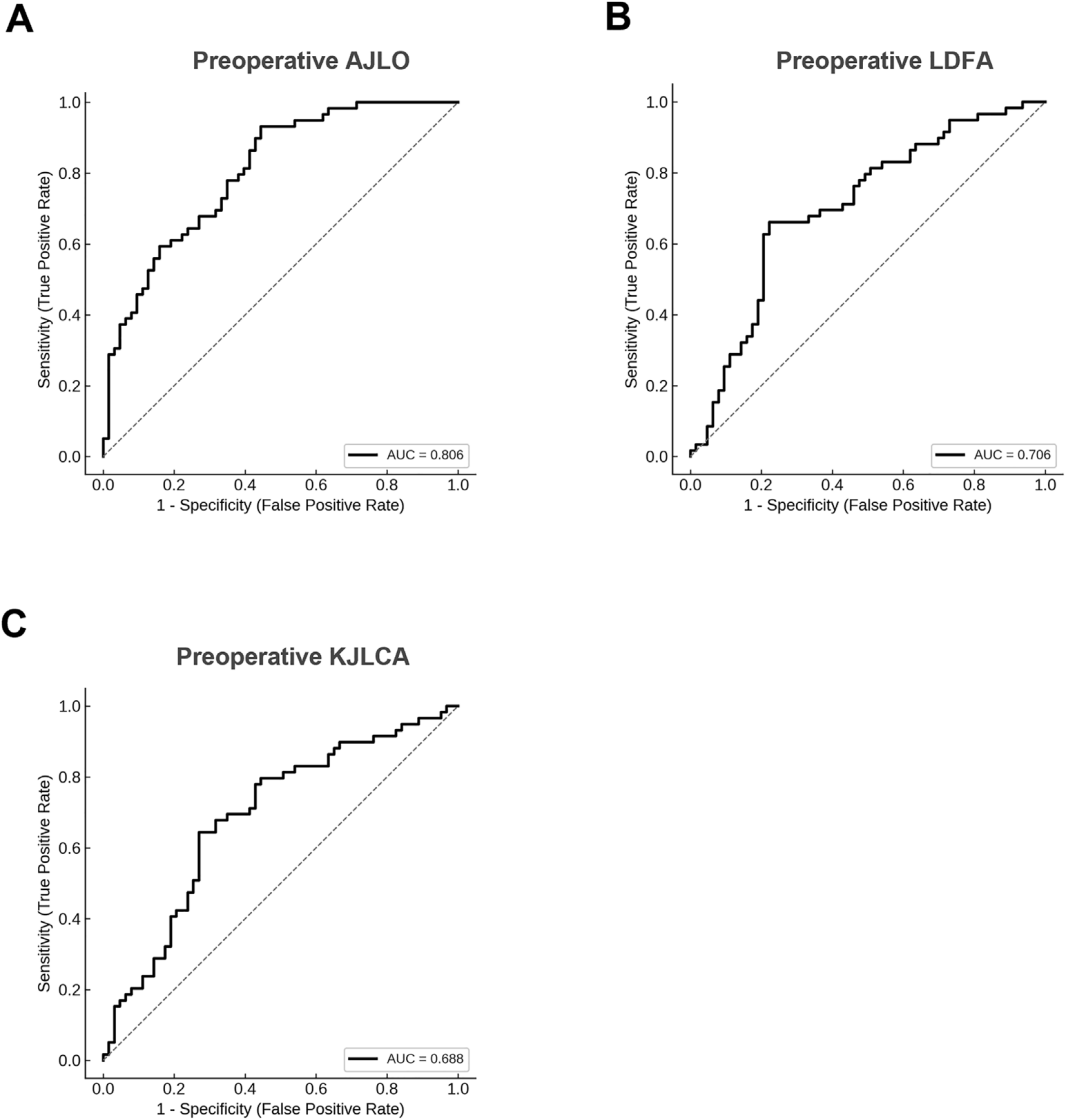
Receiver-operating characteristic curves of preoperative radiographic parameters for predicting postoperative knee joint line obliquity ≥5° in medial proximal tibial angle ≥95° cases after opening wedge high tibial osteotomy. (A) Preoperative ankle joint line obliquity (AJLO), area under the curve (AUC) = 0.806, cut-off value = 5.6°. (B) Preoperative lateral distal femoral angle (LDFA), AUC = 0.706, cut-off value = 88.9°. (C) Preoperative knee joint line convergency angle (KJLCA), AUC = 0.688, cut-off value = 4.5°.

ROC curve analyses were performed to determine the cutoff values of preoperative parameters predictive of postoperative KJLO ≥5° in knees with MPTA ≥95° after OWHTO (Figure 3). Of the factors examined, preoperative AJLO showed the highest discriminative ability, with an area under the curve (AUC) of 0.806 with the optimal cutoff value of 5.6° according to the Youden index. Preoperative LDFA and KJLCA also demonstrated moderate predictive abilities, with AUC values of 0.706 and 0.688 at cutoff values of 88.9° and 4.5°, respectively. In the subgroup analysis of knees with postoperative MPTA ≥95°, the knees with preoperative AJLO ≥5.6° had a significantly higher rate of postoperative KJLO ≥5° than the knees with preoperative AJLO <5.6° (65.1% vs 10.3%, respectively, *p* < 0.001). Similarly, the knees with preoperative LDFA ≥88.9° had a significantly higher rate of postoperative KJLO ≥5° than the knees with preoperative LDFA <88.9° (71.7% vs 28.9%, respectively, *p* < 0.001), and the knees with preoperative KJLCA ≥4.5° had a significantly higher rate of postoperative KJLO ≥5° than the knees with preoperative KJLCA <4.5° (68.5% vs 30.8%, respectively, *p* < 0.001). These findings indicate that greater preoperative AJLO, LDFA, and KJLCA are associated with an increased risk of postoperative KJLO ≥5° in knees with MPTA >95° after OWHTO.

## Discussion

The most important finding of this study was that MPTA was positively correlated with KJLO after OWHTO, but more than half of the knees with MPTA ≥95° had KJLO <5°. This finding suggests that overcorrection of the proximal tibia does not necessarily result in excessive joint line inclination. Furthermore, preoperative radiographic parameters, including the AJLO, LDFA, and KJLCA, were identified as predictors of postoperative KJLO ≥5° in the overcorrected knees with MPTA ≥95° that underwent OWHTO.

Previous studies have reported that excessive valgus correction with MPTA ≥95° may lead to an increased lateral inclination of the knee joint line, potentially causing abnormal shear forces on the articular cartilage [7] and suboptimal clinical outcomes [4, 6, 13]. However, other reports have indicated that such overcorrection does not always lead to poor results [11, 14], likely due to compensatory mechanisms in the hip and ankle joints that mitigate the increase in KJLO [9, 15]. The present study supports this interpretation by demonstrating that 52.5% of the knees with postoperative MPTA ≥95° maintained KJLO <5°. Previous studies demonstrated that valgus correction increases KJLO in proportion to MPTA, but compensatory ankle valgization or hip adduction can partly counterbalance this change [9, 15]. A recent study showed that lateral knee laxity, defined by a preoperative KJLCA >3°, was associated with increased KJLO after OWHTO [16]. Another recent study highlighted that alignment types defined by the coronal plane alignment of the knee (CPAK) classification affect how the lower limb responds to knee osteotomy and showed that arithmetic joint line obliquity, calculated by MPTA and LDFA, and LDFA were radiographic risk factors for excessive KJLO after isolated HTO [17]. These findings emphasize that, although MPTA is an important determinant of KJLO, the relationship is not linear, and postoperative joint line inclination is affected by the overall coronal alignment of the lower limb rather than by tibial correction alone.

This study specifically focused on knees with postoperative MPTA ≥95° to clarify the factors leading to excessive postoperative KJLO after overcorrection. Within this subgroup, preoperative radiographic parameters such as AJLO, LDFA, and KJLCA were independently associated with postoperative KJLO ≥5°. ROC curve analysis identified cutoff values of 5.6°, 88.9°, and 4.5° for AJLO, LDFA, and KJLCA, respectively, suggesting that preexisting coronal malalignment across the knee and ankle joints predisposes the limb to postoperative joint line inclination. Of these parameters, greater preoperative AJLO (≥5.6°) was the strongest predictor of postoperative KJLO ≥5°, indicating that a valgus-oriented ankle amplifies the lateral inclination of the tibial Plateau in the overcorrected knee with MPTA ≥95°. Conversely, an increased KJLCA (≥4.5°) reflects medial soft-tissue laxity, allowing greater lateral opening during osteotomy and, thus, contributing to joint line elevation. In addition, a larger LDFA (≥88.9°), representing relative femoral varus alignment, was also associated with postoperative KJLO ≥5°, suggesting that a more varus-oriented distal femur increases the likelihood of a laterally tilted joint line after osteotomy. These three parameters act at different anatomical levels.

From a clinical standpoint, the present findings suggest that even with postoperative MPTA ≥95°, approximately half of the cases can be successfully managed with a single-level osteotomy without developing excessive joint line obliquity. This indicates that overcorrection at the tibial level alone does not necessarily compromise coronal balance if preoperative alignment conditions are good. It also implies that, in actual clinical settings, the proportion of cases that can be adequately corrected by single-level osteotomy is greater than the proportion predicted to require double-level osteotomy based on preoperative simulation using weight-bearing radiographs [18, 19]. However, these findings also have important implications for surgical planning. When preoperative parameters exceed the identified thresholds (AJLO ≥5.6°, LDFA ≥88.9°, and KJLCA ≥4.5°), surgeons should anticipate an increased risk of postoperative KJLO ≥5° if correction is achieved at the tibial level alone. In such cases, surgical planning should include consideration of additional correction at the femoral level, such as double-level osteotomy, to distribute alignment changes more physiologically between the femur and tibia, thereby minimizing excessive joint line inclination and reducing stress concentration in the lateral compartment [20].

Several limitations should be noted. First, this was a retrospective study, and clinical outcomes were not analyzed in relation to radiographic findings; thus, the clinical implications of excessive KJLO remain to be validated. Second, the analysis was confined to coronal alignment parameters and did not account for sagittal or rotational factors that may affect load distribution. Third, radiographs were obtained under static standing conditions and may not reflect dynamic compensation during gait. Joint line orientation during functional activities may differ from static measurements, and excessive KJLO observed on standing radiographs does not necessarily translate into abnormal load distribution during walking. Future prospective studies incorporating gait analysis or other dynamic functional assessments are warranted to clarify the clinical relevance of the proposed predictive model.

## Conclusions

Although postoperative MPTA was positively correlated with KJLO after OWHTO, more than half of the knees with MPTA ≥95° maintained KJLO <5°. Thus, overcorrection of the proximal tibia does not necessarily lead to excessive joint line inclination. Preoperative AJLO, LDFA, and KJLCA are key predictors of postoperative KJLO ≥5° in overcorrected knees with MPTA ≥95°. Therefore, a comprehensive assessment of preoperative alignment, including AJLO, LDFA, and KJLCA, is essential to mitigate the risk of excessive KJLO in cases where postoperative MPTA ≥95° is anticipated.

## Data Availability

Available upon request from the corresponding author.
